# Comment on ‘How the evolution of multicellularity set the stage for cancer’

**DOI:** 10.1038/s41416-018-0091-0

**Published:** 2018-05-14

**Authors:** Pascal Jézéquel, Mario Campone

**Affiliations:** 1 Unité de Bioinfomique, Institut de Cancérologie de l’Ouest—René Gauducheau, Bd J. Monod, 44805 Saint Herblain Cedex, France; 2 INSERM U892, IRT-UN, 8 Quai Moncousu, 44007 Nantes Cedex, France; 3 Service d’Oncologie Médicale, Institut de Cancérologie de l’Ouest—René Gauducheau, Bd J. Monod, 44805 Saint Herblain Cedex, France

**Keywords:** Cancer models, Evolutionary genetics

We read the paper “How the evolution of multicellularity set the stage for cancer” with interest, which was published in a recent issue of the *British Journal of Cancer*.^1^ In this paper, the authors underlined that disruption of gene regulatory networks, which maintain the multicellular state, induces cancer. The atavistic model of cancer is undoubtedly effective in integrating many parameters in a performing heuristic framework. It hypothesises that cancer results from a transition from multicellularity to unicellularity, through an active constrained process. In this schema, dysregulation of a set of fundamental points of vulnerability, which govern multicellularity maintenance, is sufficient to model carcinogenesis.

First, an important clarification needs to be made about the cellular state of cancer cells. A tumour is a heterogeneous community of cancerous and non-cancerous cells whose global behaviour depends on numerous social-ecological and parasitic interactions. In other words, the atavistic model should emphasise that tumours represent a pseudo-multicellular neotissue, rather than a collection of unicellular tumour cells. Indeed, it can be considered as a kind of biofilm, whose life and fate depend on Darwinian and ecological principles. Contrary to unicellular or multicellular organisms, pseudo-multicellularity does not refer to a single organism but to a community of organisms, which display multicellular biological traits. These features preceded multicellularity and some of them prepared the major evolutionary transition, which lead to true multicellularity. It is important to insist on the pseudo-multicellular mode of life, because ever since the emergence of unicellular life, biofilms are the rule, rather than the exception.^2^ The 3430-million-year-old stromatolites found in Pilbara Craton in Australia indicated an early appearance of pseudo-multicellularity.^3^ The conventional scenario for the major evolutionary transition to a multicellular organism probably follows the three-state transformation series: social (pseudo-multicellular) prokaryote that evolved to social (pseudo-multicellular) protist, which evolved to a multicellular organism.^4^ Thus, backward evolution would certainly transform a “multicellular” cell into a “pseudo-multicellular” cell. Within a solid tumour only single migrating cells, which evade anoikis, can be considered as pure “unicellular” cells.

Genomic phylostratigraphy has shown that many genes considered as specific of multicellular organisms and are pointed out by the atavistic model were already present in unicellular organisms before metazoan appearance.^5^ Among these genes, we find genes involved in cell death, adherence, and tight and gap junctions. This fact clearly underlines the importance and primacy of the unicellular social mode (pseudo-multicellularity) throughout natural evolution. Genes involved in cancer have pseudo-multicellular and multicellular evolutionary origins rather than pure unicellular evolutionary origin.

Ceaseless proliferation is the most characteristic feature of cancer. But, this behaviour is rarely adopted by unicellular organisms in nature. In addition to cell communication, cell-to-substrate and cell-to-cell adhesions, earlier unicellular organisms (prokaryotes and protists) acquired a variety of anti-proliferative capabilities (cell cycle negative regulation, programmed cell death, contact-dependent inhibition, toxin-antitoxin, etc.) through the pseudo-multicellular mode of life and responses to selective pressure.^6,7^ Indeed, exponential growth may lead to species extinction due to starvation or destruction of the protective biofilm. Earlier prokaryotes inevitably faced this problem and natural evolution proposed different solutions to circumvent them, which were thereafter fixed by heredity.

Trigos et al.^1^ underlined that many hallmarks of the malignant phenotype of cancer can be interpreted as resulting from dysregulation of genes and cellular processes that appeared during transition from unicellularity to multicellularity.^8^ As previously mentioned, numerous genes involved in cancer and multicellular maintenance appeared before this transition.^5^ These genes certainly appeared in response to selective advantages conferred by the pseudo-multicellular mode of life. These advantages can be briefly summarised as protection from a wide range of environmental challenges. Second, and this is an important question, how can the atavistic model explain the induction of angiogenesis, lymphangiogenesis, axonogenesis, inflammation and immune-suppressing cell recruitment, which are frequently encountered in tumours? In these cases, the “tumours are wounds that do not heal” model seems to perform well.^9^ Third, high-throughput sequencing technology revealed that we only know half of genes involved in cancer and that these new genes could not be classified using classical cancer hallmarks categories.^10^

In conclusion, the atavistic model, which rightly focuses on dysregulation and/or loss of multicellularity-associated constraints, undoubtedly represents a powerful model, but it does not take into account other important hallmarks of cancer. It only models a part of the initiation process of carcinogenesis. In addition to what has been said above, ecological (cooperation, competition, and predation) and parasitic interactions between cells should be included in a global model of cancer.
